# Newborn Screening for Congenital Adrenal Hyperplasia: Review of Factors Affecting Screening Accuracy

**DOI:** 10.3390/ijns6030067

**Published:** 2020-08-23

**Authors:** Patrice K. Held, Ian M. Bird, Natasha L. Heather

**Affiliations:** 1 Wisconsin State Laboratory of Hygiene, University of Wisconsin School of Medicine and Public Health, Madison, WI 53706, USA; 2 Department of Pediatrics, University of Wisconsin School of Medicine and Public Health, Madison, WI 53706, USA; 3 Department of Obstetrics and Gynecology, University of Wisconsin School of Medicine and Public Health, Madison, WI 53715, USA; imbird@wisc.edu; 4 Newborn Screening, LabPlus, Auckland City Hospital, Auckland 1023, New Zealand; NHeather@adhb.govt.nz; 5 Liggins Institute, University of Auckland, Auckland 1010, New Zealand

**Keywords:** congenital adrenal hyperplasia, newborn screening

## Abstract

Newborn screening for 21-hydroxylase deficiency (21OHD), the most common form of congenital adrenal hyperplasia, has been performed routinely in the United States and other countries for over 20 years. Screening provides the opportunity for early detection and treatment of patients with 21OHD, preventing salt-wasting crisis during the first weeks of life. However, current first-tier screening methodologies lack specificity, leading to a large number of false positive cases, and adequate sensitivity to detect all cases of classic 21OHD that would benefit from treatment. This review summarizes the pathology of 21OHD and also the key stages of fetal hypothalamic-pituitary-adrenal axis development and adrenal steroidogenesis that contribute to limitations in screening accuracy. Factors leading to both false positive and false negative results are highlighted, along with specimen collection best practices used by laboratories in the United States and worldwide. This comprehensive review provides context and insight into the limitations of newborn screening for 21OHD for laboratorians, primary care physicians, and endocrinologists.

## 1. Introduction

Congenital adrenal hyperplasia (CAH) refers to a group of inherited genetic disorders caused by specific enzyme defects within the biosynthetic pathway of glucocorticoids. 21-hydroxylase deficiency (21OHD) is the most common cause of CAH, accounting for over 95% of all cases [1].

Newborns with untreated severe 21OHD, referred to as salt-wasting CAH (SW-CAH), develop progressive salt-wasting crisis during the first weeks of life, resulting in significant morbidity and mortality [2]. Newborn screening for SW-CAH provides the opportunity for early detection and treatment and has been implemented in the United States and more than 35 countries [3]. However, at present, first-tier screening methodologies lack specificity and adequate sensitivity to identify all newborns with 21OHD that would benefit from early treatment [1].

Here, we provide an overview of CAH due to 21OHD, focusing on disease presentation, pathology, genetics, diagnosis, and treatment. Next, we review the development stages of the fetal hypothalamic-pituitary-adrenal axis and adrenal steroidogenesis, so that the reader can better understand the biological processes that contribute to false positive and false negative results in newborn screening. Lastly, we will summarize different screening algorithms and approaches to specimen collection used by laboratories in the United States and worldwide to enhance detection of newborns with 21OHD.

## 2. Features of 21-Hydroxylase Deficiency

### 2.1. Clinical Manifestations

Patients with 21OHD have a range of clinical presentations which are categorized into three groups: classic salt-wasting (SW-CAH), classic simple-virilizing (SV-CAH), and non-classic (NC-CAH). The combined estimated incidence of classic 21OHD is 1 in 14,000 to 18,000 live births, with approximately 75% classified as SW-CAH and 25% classified as SV-CAH. NC-CAH is more common than the classic forms, with an estimated incidence of 1 in 200 [1].

Patients with the most severe form, SW-CAH, have <2% 21-hydroxylase activity and are unable to produce adequate amounts (if any) of cortisol and aldosterone. A salt-wasting crisis can be evident within the first 5 days of life and is characterized by progressive hyponatremia, hyperkalemia, dehydration, alkalosis, and failure to thrive, leading to shock and ultimately death, if left untreated. Patients with SV-CAH have slightly higher residual enzyme activity, as compared to the SW-CAH patients, and can generally secrete adequate amounts of aldosterone to maintain sodium balance and prevent a salt-wasting crisis [1,4].

Excess adrenal androgen production is an additional feature of 21OHD. Prenatal virilization occurs in both SW-CAH and SV-CAH and can present clinically as ambiguous genitalia in females. However, the degree of genital virilization is variable and detection is reliant on clinical acumen, such that females are at times missed or misidentified as males [5,6]. Males with 21OHD are less readily detected on clinical examination and therefore are more likely to benefit from newborn screening. Of note, data from the Sweden screening program suggests that clinical detection of classic 21OHD may be similarly unreliable for both genders [7]. Males and females with SV-CAH, who are not identified in the neonatal period, typically present in early childhood with premature development of pubic hair and rapid skeletal growth [4].

Patients with NC-CAH have up to 50% of normal 21-hydroxylase activity. The adequate amounts of cortisol and aldosterone production prevent severe clinical deficiencies. Post-natal androgen excess is variable and many patients with NC-CAH remain undiagnosed. Signs of androgen excess during childhood are less obvious and include premature adrenarche, rapid skeletal growth, and cystic acne. Common presenting features for affected adult women include hirsuitism, menstrual cycle disorders, and decreased fertility [1,4].

### 2.2. Pathology

**Figure 1:**
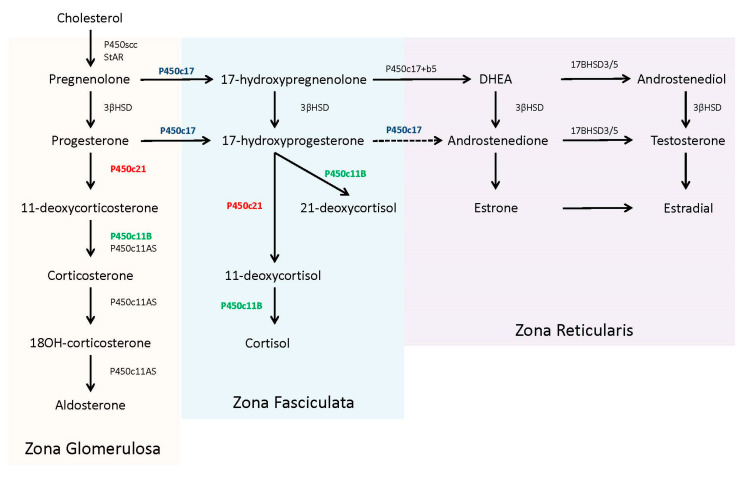
Adrenal steroidogenesis pathway. Biosynthetic pathway of mineralocorticoids (aldosterone), glucocorticoids (cortisol), and sex hormones (testosterone) within the adrenal. Partitioning for synthesis of key steroids occurring within each of the three zones (zonal glomerulosa, zona fasciculate, and zona reticularis) is provided in the shaded areas. Dashed lines with arrow heads, as compared to solid lines, denotes enzyme steps have limited affinity for conversion of the substrate to the product. DHEA = dehydroepiandrosterone, 3βHSD = 3β-hydroxysteroid dehydrogenase.

In the adrenal steroidogenic pathway, 21-hydroxylase (P450c21) catalyzes the conversion of 17-hydroxyprogesterone (17OHP) to 11-deoxycortisol to form cortisol and the conversion of progesterone to 11-deoxycorticosterone to form aldosterone (P450c21, in red, Figure 1). The required levels of cortisol (µg/dL, nmol/L range) far exceeds aldosterone (ng/dL, pmol/L range) and thereby the presence of only minimal 21-hydroxylase activity (1–2%) can produce adequate amounts of aldosterone to prevent a salt-wasting crisis [8].

Patients with 21OHD have reduced or absent production of cortisol. This deficiency leads to an uninhibited overproduction of pituitary adrenocorticotropic hormone (ACTH) in an attempt to stimulate adrenal steroidogenesis. Concentrations of steroid precursors, prior to the enzyme block, accumulate in response to this ACTH stimulation. The adrenal steroid precursors, pregnenolone, and 17-hydroxypregnenolone, are sequestered for the biosynthesis and ultimately overproduction of dehydroepiandrosterone (DHEA) and other androgens [4].

17OHP accumulates because human 17,20 lyase (P450c17) has limited to no capacity to convert it into androstenedione [8] (P450c17, in blue, Figure 1). The excess 17OHP is converted into androgens, specifically dihydroxytestosterone, via ‘backdoor’ pathways, or into an alternative product, 21-deoxycortisol, by 11β-hydroxylase (P450c11B) [9] (P450c11B, in green, Figure 1). Recent studies have noted the utility of 21-deoxycortisol, in addition to 17OHP, as a diagnostic marker for 21OHD [10]. 21-deoxycortisol is further converted into other 11-oxygentate androgens, which may also serve as useful biomarkers for disease identification and/or treatment management [11,12].

### 2.3. Genetics

21OHD is an autosomal recessive disorder caused by mutations in the *CYP21A2*. Interestingly, approximately 95% of the *CYP21A2* mutations are due to recombination events with its pseudogene (*CYP21P*) [13]. During mitosis, gene conversion with *CYP21P* introduces deleterious mutations into *CYP21A2*, while during meiosis, recombination events between the two genes cause deletions and creation of chimeric pseudogenes. More than 60 additional unique mutations in *CYP21A2* account for the remaining 5% of cases [13].

Adrenal P450 enzymes are controlled at the level of transcription and therefore the genotype-phenotype correlation is typically consistent. Deletions and nonsense mutations ablate enzyme activity and are most often associated with SW-CAH. Missense mutations typically yield 1–2% activity and are associated with SV-CAH. However, milder missense variants can produce 20–60% activity and be associated with NC-CAH [14]. In addition, patients may carry more than one mutation on either *CYP21A2* allele(s), leading to variances in severity within the three forms of CAH.

### 2.4. Diagnosis and Treatment

Infants with newborn screen results suggestive of 21OHD should be referred to and assessed by a pediatric endocrinologist. Elevated serum 17OHP levels can confirm 21OHD, and measurement of serum electrolytes and plasma renin activity identify newborns at risk of a salt-wasting crisis. A corticotropin stimulation test may be necessary when 17OHP levels are only mildly increased and diagnosis is unclear. Genotyping may assist with the interpretation of equivocal biochemical results and provide guidance for the genetic counseling of families [1].

Children with classic 21OHD require long-term glucocorticoid replacement treatment. The treatment goal is to suppress excess secretion of adrenal androgens using the lowest effective dose of glucocorticoid, typically hydroxycortisone, because glucocorticoid overtreatment is associated with growth suppression, weight gain and reduced bone mineral density. Infants with classic 21OHD are also treated with supplemental mineralocorticoids, typically fludrocortisone, coupled with sodium chloride replacement. Older children have reduced requirements for mineralocorticoid, although fludrocortisone may still be given as a glucocorticoid-sparing agent [1,9].

### 2.5. Newborn Screening

Newborn screening (NBS) for 21OHD was first initiated in Alaska in 1978 and became mandatory in all 50 states by 2009 [15]. The principal goal for screening is to facilitate the early detection of patients with severe 21OHD (SW-CAH), within the first week of life, to prevent the mortality and morbidity associated with salt-wasting crisis [1]. Historical publications demonstrate that screening readily detects the majority of patients with SW-CAH, while patients with SV-CAH are less reliably identified [7]. NC-CAH is not a target disease for NBS programs; however, there are reports of cases identified through screening [16].

17OHP is the primary marker used to identify newborns at risk for 21OHD. Initially, screening laboratories quantified 17OHP with a radioimmunoassay; however, this has since been replaced with a dissociation-enhanced lanthanide fluorescence immunoassay in automated systems (DELFIA). Typically, newborn screening laboratories set 17OHP cutoff values at low thresholds in attempts to detect all newborns with either form of classic 21OHD (100% screening sensitivity). However, this practice results in a large number of screened positive cases (cases in which 17OHP is elevated) of which very few are confirmed to have the disease (low positive predictive value (PPV)) [17]. 17OHP is elevated in both preterm and sick newborns, and typically males have higher 17OHP concentrations than females [7,18]. These factors have lead laboratories to adjust cutoff values based upon the baby’s sex, birth weight and/or gestational age [19,20,21,22,23,24], yet in general, the positive predictive value remains low (on average less than 10%) for the first-tier immunoassay performed on specimens collected within the first two days of life [25]. Lastly, there are reports of missed cases of severe SW-CAH, because measured 17OHP concentrations were below set thresholds [26], suggesting that even lower cutoffs may be needed for the first-tier assay [27]. Of note, direct comparison of published laboratory screening algorithms using PPV can be challenging due to differences in unique protocols and definitions of screened positive cases. A summary of current testing algorithms and specimen collection practices, which have improved accuracy of the screen, is presented in Section 5.

Over the past 15 years, newborn screening programs have increasingly used second-tier biochemical testing, in addition to the first-tier immunoassay, to improve specificity. This practice of two-tier testing was endorsed in the 2018 Endocrine Society Clinical Practice Guidelines [1]. The second-tier assay, performed on the original specimen, quantifies 17OHP plus additional steroids, including cortisol, 21-deoxycortisol, and androstenedione, using liquid chromatography tandem mass spectrometry. Various combinations of individual steroid concentrations and ratios have been demonstrated to enhance screening specificity, decreasing false positive rates by as much as 90% [28,29]. In a recent prospective study, a reported PPV of 17% was achieved with a screening algorithm that included both first-tier immunoassay and second-tier steroid profiling [30]. Several laboratories have also shown increased specificity after implementing molecular analysis as a second-tier test; however, not all mutations can be reliably detected in a screening setting, limiting the assay sensitivity [31,32]. To date, second-tier molecular analysis has not been implemented in routine screening.

## 3. Fetal Hypothalamic-Pituitary-Adrenal Axis and Adrenal Steroidogenesis

In this section, we outline normal fetal development and the initiation of independent adrenal steroidogenesis, highlighting how changes in maternal and placental signaling hormones effect production of fetal cortisol and other steroids, including 17OHP. This detailed analysis will set a context for later sections in which the reasons for both false positive and false negative screening results are explored.

### 3.1. Function of the HPA Axis

The hypothalamic-pituitary-adrenal (HPA) axis is the primary regulator of homeostasis within the body, managing resources during resting states, periods of high activity, and extreme stress. As summarized in the review by Howland et al., the stress stimulated cascade of hormone release begins with the production of corticotropin-releasing hormone (CRH) within neurons of the hypothalamus. Secreted CRH binds to receptors within the anterior pituitary and stimulates the release of adrenocorticotropic hormone (ACTH) [33]. ACTH then activates the steroidogenesis pathways in all zones within the adrenal cortex to maintain P450 expression and produce mineralocorticoids, glucocorticoids, and sex hormones. The newly synthesized glucocorticoid, cortisol, activates metabolism, and utilization of energy stores, regulates blood pressure, reduces inflammation, and enhances memory and attention. The stress response is terminated when excess circulating cortisol acts on glucocorticoid receptors in the hypothalamus and pituitary, inhibiting release of CRH and ACTH, respectively [33].

### 3.2. Adrenal Steroidogenesis

Unesterified cholesterol, derived from circulating low-density lipoprotein, is the precursor for adrenal steroidogenesis. The steroidogenic acute regulatory protein (StAR) assists in the transport of cholesterol from the cytosol to the inner mitochondria membrane to be converted into pregnenolone using the P450 side-chain cleavage (P450scc) enzyme. Pregnenolone then enters the smooth endoplasmic reticulum, where it is further converted to specific steroids, depending upon the compliment of other required enzymes [4]. The zona fasciculate of the adult adrenal contains abundant 17α-hydroxylase activity (P450c17), along with 3β-hydroxysteroid dehydrogenase (3βHSD), 21-hydroxylase (P450c21), and 11β-hydroxylase (P450c11B), which are all necessary for the production of cortisol from pregnenolone. The zona glomerulosa lacks the 17α-hydroxylase enzyme, committing pregnenolone to the exclusive production of aldosterone. In contrast, the zona reticularis in the adult adrenal contains 17α-hydroxylase and abundant cytochrome b5, which supports 17,20 lyase activity by the P450c17 protein. Of note, human 17,20 lyase activity favors production of dehydroepiandrosterone (DHEA), not androstenedione, which is rapidly used by other tissues for testosterone production [4,10]. The complete steroidogenic pathway with partitioning for each zone is shown in Figure 1.

### 3.3. Maternal, Placental, and Fetal Unit

**Figure 2:**
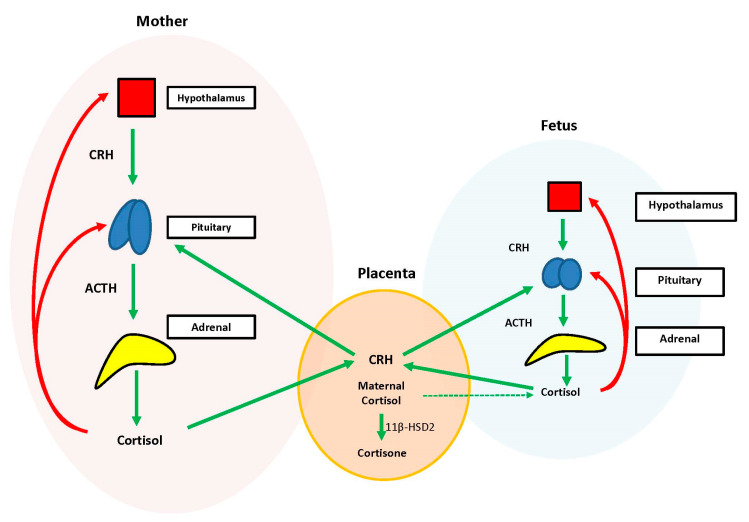
Maternal, placental, fetal unit. A representation of the hormone exchange between the maternal, fetal, and placental units. The maternal hypothalamic-pituitary-adrenal (HPA) axis stimulates cortisol production. Maternal cortisol is transferred to the placenta and converted into cortisone by 11β-HSD2, with only minimal exposure of the fetus to cortisol (dashed green line). Maternal cortisol also stimulates production of placental corticotropin-releasing hormone (CRH), which in turn activates synthesis and release of additional maternal adrenocorticotropic hormone (ACTH), creating a positive feedback loop (green arrows). Placental CRH also enters the fetal circulation and stimulates release of ACTH, which allows for production of fetal cortisol. Fetal cortisol stimulates additional placental CRH production creating a second positive feedback loop. Excess cortisol inhibits production of CRH and ACTH both within the maternal and fetal units (red arrows). Figure was modified from the Howland et al. (2017) reference [33].

During the prenatal period, the placenta acts at the interface between the maternal and fetal compartments, producing hormones that adjust maternal physiology to benefit mother and baby. Placental CRH, expressed by week 7 of gestation, is identical to maternal CRH, and functions to co-regulate stress hormone production during pregnancy. Specifically, placental CRH stimulates synthesis and release of maternal ACTH and downstream production of maternal cortisol from the adrenals. In a positive feedback loop, maternal cortisol then stimulates production of additional placental CRH (Figure 2) [33].

During the prenatal period, the structures of the fetal HPA axis are undergoing tremendous growth, but the independent sequential release of fetal CRH, ACTH, and cortisol is not established until the third trimester [33]. The fetal stress response relies heavily upon the input of maternal and placental hormones. Placental CRH enters the fetal circulation and directly stimulates the fetal pituitary to release ACTH and the fetal adrenal to increase overall responsiveness to ACTH. This allows for production of systemic fetal cortisol as early as 24 weeks gestation. Fetal cortisol stimulates additional placental CRH production, similar to maternal cortisol, creating a second positive feedback loop (Figure 2) [33].

**Figure 3:**
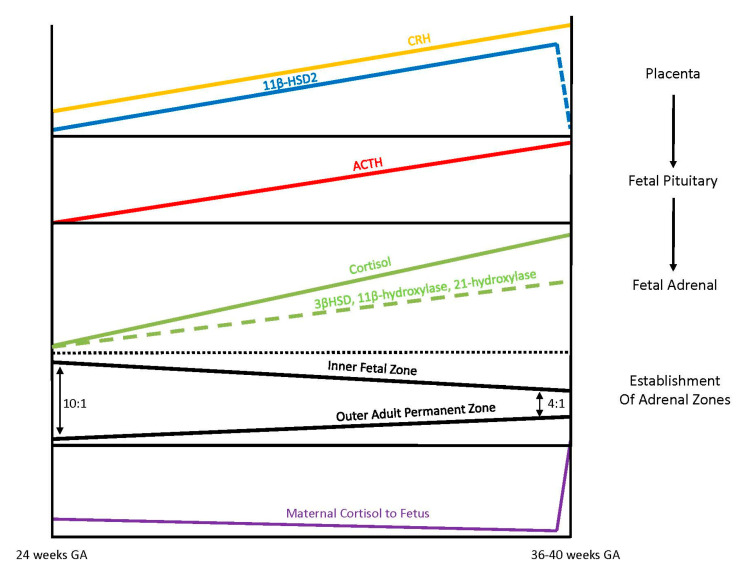
Dynamic changes of the fetal hypothalamic-pituitary-adrenal axis. During the third trimester, 24 to 36–40 weeks of gestation, the placenta increases production of corticotropin-releasing hormone (CRH). In response to placental CRH, production of adrenocorticotropic hormone (ACTH) by the fetal pituitary accelerates and stimulates the fetal adrenal to undergo steroidogenesis for cortisol production. Required enzymes for cortisol production within the adrenal, 3β- hydroxysteroid dehydrogenase (3βHSD), 21-hydroxylase (P450c21), 11β-hydroxylase, are activated. The establishment of the outer adult permanent zone of the adrenal also occurs during the third trimester. During the third trimester, activity of 11β-hydroxysteroid dehydrogenase 2 (11β-HSD2) within the placenta also increases and thereby exposure of the fetus to maternal cortisol remains low until the surge right before delivery. Figure was modified from the Howland et al. (2017) reference [33].

Exposure of the fetus to the exponential output of maternal cortisol is moderated by the placenta because excess maternal cortisol can otherwise inhibit fetal pituitary ACTH release. Specifically, the placental enzyme 11β-hydroxysteroid dehydrogenase 2 (11β-HSD2) oxidizes the biologically active maternal cortisol into its inactive form, cortisone [34], such that only a minimal amount of maternal cortisol will enter fetal circulation. However, by 34–35 weeks gestation, the activity of placental 11β-HSD2 decreases, allowing a surge of maternal cortisol into fetal compartments, which facilitates fetal organ maturation and enhances neurodevelopment prior to delivery [33,35] (Figure 3).

### 3.4. Fetal Adrenal Steroidogenesis

The fetal adrenal cortex is one of the most vascularized and active organs in early gestation, consisting of both an inner fetal zone and the outer adult or permanent zone [33]. In term infants, the ratio of the fetal zone to the adult zone is about 4 to 1, but the ratio is increased with lower gestational ages because the permanent zone matures later in response to the fetal pituitary hormone ACTH production [36]. The inner fetal zone originates in early development with the main function to produce DHEA, a precursor of estrogen, which is critically important for maternal adaption to pregnancy. For this reason, early fetal adrenals have low expression of 3β-HSD diverting all steroid precursors to DHEA [37,38]. However, from 24 weeks onward, fetal 3β-HSD activity in the permanent zone begins to increase, allowing independent synthesis of cortisol. By term, 75% of cortisol in fetal circulation is of fetal origin [33,39]. Taken together, these different stages of fetal adrenal development suggest that the abundance of steroidogenic precursors and products may differ between preterm and full-term newborns (Figure 3).

### 3.5. Preterm and Sick Infants

Multiple studies have demonstrated that basal cortisol concentrations in preterm and full-term newborns are comparable. Interestingly, the cortisol levels in both sick and healthy preterm newborns are also similar [40,41]. While cortisol levels would be anticipated to increase in preterm and sick newborns due to a stress response, this does not appear to be the case and it is possible that appropriate mechanisms to initiate a stress response are lacking. For cortisol synthesis to occur in response to stress, two actions are required. The fetal HPA axis must be intact to recognize stress and be capable of signal release of CRH by the hypothalamus and ACTH by the pituitary. Second, in response to ACTH stimulation, the adrenal enzymes must be present and functional to facilitate steroidogenesis. Several studies have evaluated both of these two required components to determine the underlying cause of reduced cortisol production in preterm newborns.

An early study investigated the function of the HPA axis in extremely low birth weight, stressed, premature infants. The infants were stimulated with either ovine CRH or ACTH and an appropriate ACTH or cortisol response was observed, respectively. As judged by the ability to respond to hormone stimulation, it was suggested that the pituitary and adrenal portions of the HPA axis appear to be intact as early as 26 weeks gestation [41]. A later study, evaluating preterm infants less than 32 weeks gestation age, confirmed the reports of previous study, demonstrating appropriate response to stressful stimuli by the elevation of ACTH [42]. These findings demonstrate a highly responsive HPA axis at early stages of the third trimester.

Other studies have directly evaluated adrenal steroidogenesis in full term newborns as compared to both premature healthy and sick newborns. The 17OHP, 11-deoxycortisol, and aldosterone concentrations were higher in sick preterm infants than in healthy preterm infants. Additionally, compared to full term newborns, preterm infants had significantly higher 17-hydroxypregnenolone, 17OHP, and DHEA sulfate concentrations. Cortisol and aldosterone values were not different between the sick and healthy preterm infants and were similar to concentrations measured in full term infants [43]. It was hypothesized from this data that adrenal enzyme activity in both healthy and sick preterm newborns is inadequate for production of cortisol. Further studies assessing enzyme function determined that 3β-HSD activity was not significantly reduced in extremely premature neonates, but activity of 11β-hydroxylase was markedly reduced [38]. Additional studies suggest that the activity of 21-hydroxylase is also decreased [36]. All authors concluded that very low birth weight premature infants are unable to mount a cortisol response due to deficiencies in multiple enzymes [44]. Accumulation of these steroid precursors, namely 17-hydroxypregnenolone and 17OHP, due to the decreased activity of successive enzymes, leads to a distribution of steroid concentrations that can actually mimic patterns observed in patients with 21-hydroxylase deficiency.

Throughout the third trimester, the adrenal continues to mature in response to pituitary activation and the permanent zone continues to delineate into three functional adult zones. This maturation process allows steroidogenic enzyme activities within the three zones to increase, resolving the back-up of steroid precursors, and allowing for independent cortisol synthesis at birth.

### 3.6. Effects of Glucocorticoid Treatment

Synthetic glucocorticoid administration is the standard of care for pregnant women at risk for premature delivery. This treatment promotes maturation of fetal organ systems to prepare the fetus for extra-uterine life however, excessive glucocorticoid exposure may have adverse effects on the fetus, including transient suppression of HPA activity [35,45]. Synthetic glucocorticoids pass through the placenta without being metabolized by 11β-HSD. Several studies showed that prenatal administration of synthetic glucocorticoids resulted in decreased cortisol production within the newborn for up to one week or longer after delivery [36,46]. Another study substantiated earlier reports by demonstrating that betamethasone blocked ACTH stimulation, leading to decreased expression of key steroid biosynthetic enzymes, P450scc, and 17-hydroxylase [45]. Depressed basal cortisol levels were also measured in newborns receiving dexamethasone or betamethasone treatment, and remained low for up to 7 days [40]. In general, however, the immediate and long-lasting impact of synthetic glucocorticoid administration on adrenal function in the newborn remains unclear and additional studies are needed to provide further clarification [32,42,46].

## 4. Review of False Positives and Negatives

In this next section we draw on what is known about the pathology of 21OHD, fetal HPA axis development, and adrenal steroidogenesis to explore reasons why both false positives and false negatives occur in newborn screening.

### 4.1. False Positives

It has been well documented by many laboratories that the first-tier immunoassay used to measure 17OHP as an indicator of 21OHD generates a large number of false positive cases, leading to a low positive predictive value for the first-tier screen (estimated to be less than 10%) [25]. From our assessment, false positives can be attributed to five potential sources, which are outlined below.

#### 4.1.1. Physiological Changes in 17OHP Concentrations after Birth

The delivery of a newborn is a stressful event that causes elevations of multiple steroids, including 17OHP. 17OHP concentrations are higher in newborns right after birth and slowly decrease over a 24–48 h period. As demonstrated in an early report, median blood 17OHP concentrations in healthy term infants were greater than 100 nmol/L in cord blood, decreasing to 38 nmol/L by 12–18 h, and to 23 nmol/L by 24 h of life [47]. The implication of this data is that screening cutoffs should be adjusted to the time of collection [23]. If screening laboratories do not account for collection times, results from early sample collection (collected prior to 48 h of life) may be falsely identified as positive.

#### 4.1.2. Immature Adrenal Function in Preterm Babies

Premature newborns produce large amounts of 17OHP due to adrenal enzyme immaturity. As outlined in the previous section, studies have demonstrated decreased activity of the 11β-hydroxylase enzyme in both healthy and sick premature infants and decreased 21-hydroxylase activity in babies born prior to 29 weeks [36,38]. Because adrenal steroidogenesis is not fully active in preterm babies, steroid precursors, including 17OHP, back up, leading to increased concentrations. All newborn screening programs should consider use of higher 17OHP cutoff values in premature babies, adjusting for gestational age and/or birthweight, to minimize false positives [21,24]. However, even with these stratifications of cutoff values, programs may still struggle with a low positive predictive value for the assay in premature newborns [7]. Section 5 below provides additional screening algorithm modifications to consider that can improve accuracy in preterm babies.

#### 4.1.3. Stress Inducing Conditions of the Mother and Newborn

The fetal HPA axis responds to stress by driving an increase in ACTH and subsequent steroidogenesis, resulting in elevated concentrations of cortisol and steroid precursors, including 17OHP. Elevated 17OHP concentrations in newborns with respiratory distress syndrome has been documented in multiple studies [48]. Other fetal conditions associated with an elevated 17OHP concentration include hydrops fetalis, intraventricular hemorrhage, septicemia, congenital pneumonia, perinatal asphyxia, major abdominal surgical conditions, and severe congenital heart disease [49]. In mothers with pre-eclampsia, mean infant 17OHP levels were elevated and found to be associated with the presence of intrauterine growth restriction [50]. Therefore, fetal and maternal conditions can increased likelihood for a false positive screen. Physicians and laboratorians may wish to consider these complications when interpreting screening results.

#### 4.1.4. Laboratory Methodologies

Performance of the 17OHP screening immunoassay is limited by antibody cross reactivity specifically with 17-hydroxypregnenolone sulfate, the major interfering compound [51]. This steroid is also found at higher concentrations in premature infants, as described in Section 3 above, contributing to the large number of false positive screens within premature babies. Immunoassay specificity can be improved with an initial extraction to remove cross-reacting steroid sulfates [52]. Measurement of 17OHP concentration by liquid chromatography-tandem mass spectrometry provides additional specificity, particularly among preterm infants [53]. Laboratories should consider use of a two-tier testing strategy to increase the accuracy of the screen.

#### 4.1.5. Other Forms of CAH

It is important to mention that other forms of CAH cause elevations of 17OHP. Although significantly less common, accounting for fewer than 5% of all cases, CAH due to 11-hydroxylase deficiency, 3β-HSD deficiency, and P450 oxidoreductase deficiency can all present with elevated 17OHP levels [10]. Physicians and screening laboratories should be aware of the differential diagnosis for an elevated 17OHP.

### 4.2. False Negatives

In general, patients with severe 21OHD (SW-CAH) are readily detected by screening programs, while patients with simple-virilizing CAH, are less reliably identified. However, there are also reports of screening laboratories that have missed SW-CAH cases [26]. Below, we have identified five potential causes for low 17OHP values that can result in false negative cases.

#### 4.2.1. Early Collection of Newborn Screening Specimen

17OHP values increase over time in untreated newborns affected with 21OHD [16,17,54]. Recent studies published by programs in the United States who routinely perform two newborn screens, have documented cases of both classic CAH (SW-CAH and SV-CAH) and NC-CAH identified on the second screen, due to late rising 17OHP concentrations [55,56,57]. Thus, screening accuracy for classic 21OHD, in particular for SV-CAH, may be relatively poor within the first 2 days of life when many screening laboratories collect specimens, increasing the risk of missed cases. Collection of a second screen could be considered as a means to improve screening accuracy. Additional details are provided in Section 5 below.

#### 4.2.2. Immature Adrenal Function in Preterm Babies

During fetal development, the main function of the inner fetal zone of the adrenal is to produce dehydroepiandrosterone (DHEA). For this reason, early fetal adrenals have low expression of 3β-HSD, diverting all steroid precursors to production of DHEA [37,38]. However, within the third trimester, fetal 3β-HSD activity in the permanent zone begins to increase, enabling steroidogenesis and independent synthesis of cortisol [33,39]. The authors hypothesize that depending upon the stage of fetal adrenal development and the activity of the key enzyme 3β-HSD, the flux through the immature biosynthetic pathway of cortisol may not be adequate to elevate 17OHP concentrations in preterm newborns affected with 21OHD.

#### 4.2.3. Increased Fetal Exposure to Maternal Cortisol

Maternal cortisol levels increase nearly 10-fold over the course of pregnancy and the placenta’s role is to regulate fetal exposure to maternal cortisol, through the action of 11β-HSD. This regulation is incomplete and approximately 10% of maternal cortisol enters fetal circulation [33,34,35]. As described in Section 3, maternal cortisol suppresses both the production of fetal ACTH and its stimulation of steroidogenesis within the fetal adrenals. The authors hypothesize that excess maternal cortisol exposure may reduce flux through the fetal steroidogenic pathway, leading to decreased the amounts of 17OHP accumulation in patients with 21OHD, and a risk for missed cases by newborn screening.

#### 4.2.4. Glucocorticoid Treatments

Conflicting evidence has been published on the suppressive effect of antenatal glucocorticoid treatments on pituitary adrenal function. The number of doses, the concentration of a single treatment, and the timing of when the last dose was administered can likely impact the magnitude of ACTH suppression resulting in decreased steroidogenic enzyme activities and subsequent reduction of cortisol synthesis. One study demonstrated that multiple courses of synthetic steroids in preterm infants decreased the 17OHP concentrations by ~30% in filter-paper blood [58]. Glucocorticoid treatments are a risk factor for adequate screening detection, due to the temporary reduction in 17OHP concentrations.

#### 4.2.5. Mild Forms of 21-Hydroxylase Deficiency

As mentioned in previous sections, the activity of the 21-hydroxylase enzyme and the severity of the disease can be inferred from the 17OHP concentrations measured in untreated individuals. Patients with SW-CAH have the highest serum 17OHP levels, followed by patients with SV-CAH. Patients with NC-CAH have even smaller elevations, especially in the newborn period. Votava et al. estimated a false-negative rate of at least 33% in children with the moderate to mild forms of 21OHD [59]. Therefore, it should be well communicated to physicians that newborn screening is not adequate for identifying all patients with SV-CAH or NC-CAH.

## 5. Influence of Specimen Collection Times

In the previous section, we reviewed physiological factors, conditions of the newborn, and treatments that contribute to both false positive and false negative 21OHD screening results. Later specimen collection, after 48 h of life, can minimize many of the confounding factors that contribute to poor screening accuracy. However, any delay in specimen collection will also lead to a delay in diagnosis. Screening laboratories need to balance the accuracy of results with the urgency to detect newborns with 21OHD and other severe disorders on the newborn screening panel. Below is an abbreviated summary of specimen collection practices and outcomes reported by laboratories in the United States and other countries.

### 5.1. Screening in the United States

The timing of newborn screening specimen collection for programs in the United States has evolved over time in response to both changes in healthcare practices and federal recommendations. When screening began, blood specimens were typically collected between 48–96 h after birth, allowing time for key analytes to accumulate in response to dietary intake. Over time, collections began to occur earlier, even prior to 24 h of life, in response to early discharge practices within hospitals. To minimize the risk of a missed case, several states (currently 13 of the 50) incorporated a routine second screen, between 8–14 days of life, to provide a second opportunity to identify affected newborns. In 2015, the federal advisory committee on heritable disorders in newborns and children (ACHDNC) determined that, in order to facilitate timely diagnosis and treatment, the optimal time for specimen collection was between 24–48 h of life [60]. To our knowledge, all states have adopted this recommendation.

Early studies from Wisconsin and Texas reported identification of SW-CAH patients on the first screen at 1–2 days of life. However, milder forms of CAH (SV-CAH or NC-CAH) were either missed in Wisconsin, a one-screen state, or identified in Texas, a two-screen state, on the second screen [18,61]. Reports from Minnesota, Colorado, and the Northwest Regional Newborn Screening Program have also documented missed cases of both SW-CAH and SV-CAH on the first screen [26,56,57]. Colorado reported the sensitivity of their first screen as 71.8% for classic CAH (both SW-CAH and SV-CAH), with a false negative rate of 28.2% [56]. Similarly, the NWRSP stated that 25% of all confirmed cases of 21OHD were identified on the second screen. Of these infants identified on the second screen, 39% were classified as SW-CAH and 61% were classified as SV-CAH [57]. In 2015, a large multi-state comparison study found a similar detection rate for SW-CAH cases between one- and two-screen states; however, the detection rate for SV-CAH and NC-CAH was significantly higher in the two-screen states, with the majority being identified on the second screen. The study also reported missed cases of classic CAH in both one and two screen states, suggesting that delayed diagnoses may not be solely due to differences in collection practices [55].

In 2020, a nationwide US study comparing CAH screening protocols and outcomes, within a given year, was published [25]. The report included 17 states, of which 4 had a mandatory second screen. All but one of the 17 unique screening algorithms included cutoffs stratified by birthweight, and none of the states reported using a second-tier assay. The PPV ranged from 0.7% to 50% (mean 8.1%), with the two highest PPV found in two of the four states with mandatory second screen (50% and 20%). Taken together, this study, along with other historical reports, suggest that screening accuracy, defined as a high PPV, may be achieved through collection of a second specimen, along with an increased identification of milder forms of CAH (SV-CAH and NC-CAH); however, it may not eliminate the risk for missed, false negative, cases

Several states have implemented second-tier testing, using a steroid profile, for assessment of CAH (refer to Section 2.5). This practice, which is endorsed by the Endocrine Society Clinical Practice Guidelines, will also decrease false positive cases and improve the PPV. However, the majority of screening laboratories worldwide still only use a first-tier immunoassay. Additionally, second-tier testing will not address the risk for false negatives, due to the required reflex of an abnormal first-tier 17OHP concentration, as measured by the immunoassay.

### 5.2. Worldwide Screening

As of 2015, over 35 countries worldwide perform newborn screening for 21OHD with specimen collection typically occurring after 48 h of life, in contrast to the United States [3]. Below we review four recent publications from screening programs in Sweden, Israel, Brazil, and the Netherlands, and summarize how collection times have impacted screening sensitivity and the PPV of the assay. All of these programs screen for 21OHD using the 17OHP immunoassay, minimizing variability in outcomes due to differences in the screening tests.

A publication summarizing 26 years screening for 21OHD in Sweden reported 100% sensitivity for SW-CAH CAH and 80% sensitivity for SV-CAH [7]. All collections occurred after 48 h of life and cutoffs values were stratified by gestational age. The PPV of the screen was reported as 13.4% (25% for full term babies and 1.4% for preterm babies), which is significantly higher than that obtained by screening laboratories in the United States. Similarly, in a report from Israel, the screening sensitivity for classic CAH (SW-CAH and SV-CAH) was 95.4%, with an overall PPV of 16.5% [24]. Specimen collection occurred slightly earlier in this study, between 36–72 h of life, and cutoffs were stratified by both gestational age and birthweight.

Brazil published their screening algorithm in which the 17OHP cutoff levels were adjusted for the baby’s birthweight and also age at specimen collection. Stratification of the population by two sampling time points, 48 to <72 h and ≥72 h, yielded a PPV of 5.6% and 14.1%, respectively, for the screen. No SW-CAH cases were missed. Collection prior to 48 h of life was strongly discouraged by the authors [62].

In 2019, the Dutch neonatal screening program published outcomes for 21OHD screening, with sample collection occurring between days 3–7 of life [6]. There were no missed SW cases and PPV was also high at 24.7%. However, several newborns presented unwell in a salt-wasting crisis prior to the physicians obtaining results from the screening laboratory. Due to the severity and early presentation of salt-wasting crisis in affected individuals, any delay in specimen collection may increase likelihood for detrimental outcomes.

All four of these studies address factors in normal physiology, such as the changing 17OHP concentrations after birth and the effects of prematurity on adrenal steroidogenesis that lead to decreased PPV of the first-tier screening assay. Accuracy of screening appears to be better when cutoff values are adjusted for the time of collection or by delaying specimen collection until after, at minimum, 48 h of life. However, all the authors’ caution that delayed collection may lead to delayed diagnosis with the onset of symptoms prior to identification.

### 5.3. Screening in Premature Newborns

This review has detailed how adrenal immaturity, particularly in preterm newborns, contributes to reduced accuracy of 21OHD screening assays when samples are collected within the first few days of life. Many screening laboratories have observed this phenomenon and some have even proposed not screening preterm newborns because the positive predictive value of testing is too low; less than 0.4% PPV in a French report [63]. In addition, it could also be argued that preterm babies are already being monitored in a medical environment where there is minimal risk of missing a salt-wasting crisis.

Expert focus groups have recommended modified screening algorithms in premature babies to minimize the risk of both false negative and false positive screens. The Endocrine Society endorsed the Minnesota practice of rescreening premature, low birth weight infants (<1800 g) at 2 and 4 weeks of age [1,64]. Specifically, Minnesota demonstrated that timing of sample collection in premature babies played a more important role in reducing false positive results than implementation of second-tier testing [64]. Similarly, the Clinical Laboratory Standards Institute (CLSI) recommends that all preterm (<37 weeks gestation) and low birth weight (<2500 g) infants be screened on admission into the neonatal intensive care unit (NICU), and again at 48–72 h after birth, if the initial screen was performed at less than 24 h of life. A final screen is also recommended at 28 days of life [65]. Amongst the three groups, there is consensus that monitoring of 17OHP concentrations in premature newborns, 2–3 times within the first month of life, is needed to improve testing accuracy.

## 6. Conclusions

Early detection of 21OHD through newborn screening allows for treatment of most individuals prior to the onset of symptoms, preventing devastating outcomes from severe salt-wasting crises. The success of 21OHD screening can be assumed by the worldwide adoption of this disease onto screening panels. However, many programs report poor specificity with first-tier screening methods, leading to high false positive rates, and low positive predictive values. Likewise, limited sensitivity to detect all newborns with classic CAH is a significant concern.

In this review, we have summarized the pathology of 21OHD, along with the stages of fetal HPA axis development and functional adrenal steroidogenesis which contribute to reduced accuracy in screening assays. We highlight five factors that contribute to false positive cases and an additional five contributors to false negative cases. Lastly, we summarized how various approaches to specimen collection, used by screening laboratories worldwide, have impacted CAH screening performance. Adjustments for prematurity and timing of specimen collections post-delivery are major factors that must be considered in order to maximize the effectiveness of the screen.

It is hoped that this comprehensive review of key contributors to screening inaccuracies can provide insight for laboratorians, primary care physicians, and endocrinologists.
